# The Effects of Prices on Alcohol Use and its Consequences

**Published:** 2011

**Authors:** Xin Xu, Frank J. Chaloupka

**Keywords:** Alcoholic beverage, alcoholic-beverage distribution laws, alcoholic-beverage sales, alcoholic-beverage tax, alcoholic-beverage price, price elasticity, supply and demand, policy on alcoholic beverages, economic theory of alcohol and other drug (AOD) use, problematic AOD use

## Abstract

Over the past three decades, economists and others have devoted considerable effort to assessing the impact of alcoholic-beverage taxes and prices on alcohol consumption and its related adverse consequences. Federal and State excise taxes have increased only rarely and, when adjusted for inflation, have declined significantly over the years, as have overall prices for alcoholic beverages. Yet studies examining the effects of increases of monetary prices (e.g., through raising taxes) on alcohol consumption and a wide range of related behavioral and health problems have demonstrated that price increases for alcoholic beverages lead to reduced alcohol consumption, both in the general population and in certain high-risk populations, such as heavier drinkers or adolescents and young adults. These effects seem to be more pronounced in the long run than in the short run. Likewise, price increases can help reduce the risk for adverse consequences of alcohol consumption and abuse, including drinking and driving, alcohol-involved crimes, liver cirrhosis and other alcohol-related mortality, risky sexual behavior and its consequences, and poor school performance among youth. All of these findings indicate that increases in alcoholic-beverage taxes could be a highly effective option for reducing alcohol abuse and its consequences.

Over the past three decades, economists and others have devoted considerable effort to assessing the impact of alcoholic-beverage taxes and prices on alcohol consumption and its related adverse consequences. Numerous studies have examined the effects of increases in monetary prices (e.g., through raising taxes) on a wide range of behavioral and health problems related to alcohol use, including heavy drinking, drinking and driving, violence and other related crimes, liver cirrhosis mortality, suicides, reproductive issues (including risky sexual behaviors, sexually transmitted diseases, and abortions), and school performance. Some of these studies specifically have focused on high-risk populations, such as adolescents and young adults.

This article first briefly reviews trends in alcoholic-beverage excise taxes as well as the limited literature addressing the connection between taxes and prices. The majority of the article then focuses on studies investigating the effects of prices (or taxes) on alcohol use and abuse and related adverse consequences (for additional reviews, see Chaloupka 2002; Chaloupka et al. 1998, 2002; Cook and Moore 2000, 2002; Wagenaar et al. 2010). Given the size and scope of the literature in this area, this article is not intended to be an encyclopedic review but aims to summarize the general findings and highlight recent studies. Taken together, the findings confirm an inverse relationship between alcohol prices and the demand for alcohol consumption—that is, the higher the price, the lower the demand. Moreover, policies that raise alcoholic-beverage taxes and, consequently, prices are effective in reducing alcohol use and abuse as well as related health, economic, and social consequences.

## Trends in Alcoholic-Beverage Taxes and Prices

From an economic perspective, various public policies that can affect the full price of alcoholic beverages—that is, the monetary costs (i.e., prices) plus the time costs and expected legal costs associated with alcohol use—also influence alcohol use. For example, Xu and Kaestner (2010) found that the increases in weekly hours of work were inversely associated with binge drinking—that is, binge-drinking frequency declined if people had less free time. Likewise, other studies showed that minimum-drinking-age and zero-tolerance laws reduced youth alcohol consumption and driving after drinking by increasing the expected legal costs of alcohol use (Carpenter 2004; Hingson et al. 1989, 1994; Liang and Huang 2008; Saffer and Grossman 1987; Wagenaar and Toomey 2002; Wagenaar et al. 2001; Wechsler et al. 2003). Many States also implement additional policies that reduce the availability of alcoholic beverages, including limits on the places where, or times when, alcoholic beverages can be sold or dram shop laws,^1^ thus raising the time and legal costs associated with obtaining alcohol. Because other articles in the issue will discuss these policies and their impacts on alcohol consumption and related consequences in more detail, this article focuses on policies that affect the monetary prices of alcoholic beverages.

### Federal Excise Taxes on Alcoholic Beverages

**Figure 1:**
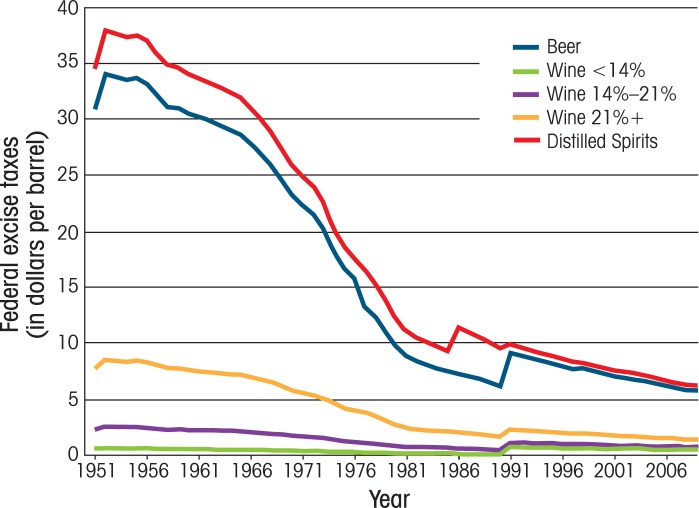
Average real Federal excise taxes (in dollars per barrel) on alcoholic beverages 1951–2009. SOURCE: U.S. Department of Treasury, Alcohol and Tobacco Tax and Trade Bureau. Historical tax rates [article online]. Available at: http//www.ttb.gov/tax_audit/94a01_4.shtml. Accessed July 28, 2011 NOTE: Current Federal excise taxes were set on January 1, 1991. Inflation-adjusted values are based on the All Urban Consumers consumer price index series.

Excise taxation is the primary policy for directly influencing the prices of alcoholic beverages. The Federal Government imposes volume taxes on distilled spirits, wine, and beer; however, increases in these taxes have been rare in recent decades. In fact, since 1951, Federal excise tax rates on beer and wine only have increased once (on January 1, 1991) and the tax on distilled spirits only twice (on October 1, 1985, and January 1, 1991). As a result of these infrequent and modest increases, the real tax rates (i.e., inflation-adjusted values) have declined significantly over the years. For example, the real Federal beer excise tax, which was nearly 31 dollars per barrel in 1951, had fallen to approximately 6 dollars per barrel in 2009. Likewise, the Federal excise tax on distilled spirits fell from 35 dollars in 1951 to 6 dollars in 2009 (see figure 1).^2^

### State Excise Taxes on Alcoholic Beverages

**Figure 2:**
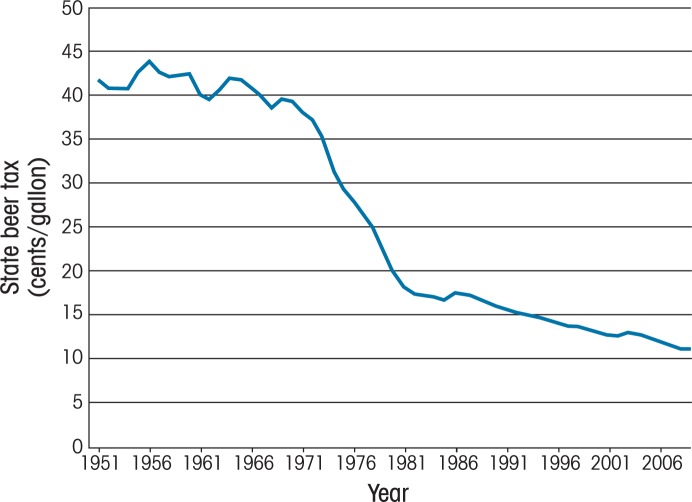
Average real State taxes on beer tax 1951–2009. SOURCE: Brewers Almanac 2009: Beer Institute. NOTE: The estimates are based on the mathematic average of beer taxes of 50 States and the District of Columbia. Inflation-adjusted values are based on the All Urban Consumers consumer price index series.

Excise-tax policies vary widely across States, with some States imposing taxes on prices (i.e., ad valorem taxes) and others levying excises on quantity or volume (i.e., specific taxes). All States impose a tax on beer; in addition, all license States also impose taxes on wine and spirits.^3^ In general, these State excise taxes are highest for distilled spirits. State excise taxes, for the most part, have followed the same patterns as Federal taxes, with only infrequent and modest increases that have resulted in substantial declines over time in the real values of these taxes. The degree to which the real value of the State taxes has dropped depends on the inflation rate and the latest tax rates imposed by a given State. More than 20 States have not raised their beer taxes for at least 20 years, and only about 10 States have raised them in the last decade.^4^ In some extreme cases, the deflated tax rates per drink have even declined to close to zero. For example, the nominal State beer excise tax in Wyoming was 2 cents per gallon in 2009, and it had been set since 1963. Similar situations exist in (but are not limited to) Missouri, Wisconsin, Oregon, and Kentucky. Estimates indicate that from 1951 to 2009, the average real State beer tax has fallen from almost 42 cents per gallon to just over 11 cents per gallon (see figure 2) (Beer Institute 2009).

### Alcohol Excise Taxes and Prices

Excise taxes create a wedge between the price that producers receive for their products and the final retail price that consumers pay. From a theoretical perspective, increases in excise taxes therefore automatically should lead to increases in the final retail prices of alcoholic beverages. The extent to which changes in excise taxes can be passed on to the final prices is an empirical question. The studies addressing this issue consistently found that in fact in the U.S. market of alcoholic beverages, an overshifting of excise taxes occurs, meaning that prices for alcoholic beverages have risen by more than the amount of the tax increases (Cook 1981; Kenkel 2005; Young and Bielinska-Kwapisz 2002). The rates by which tax increases can be passed on to final retail prices (known as the pass-through rates) range from 1.2 to 4.2, depending on specific brands and types of alcoholic beverages analyzed as well as on sales location (i.e., on premises versus off premises). Thus, the limited empirical evidence indicates that increases in alcoholic-beverage excise taxes likely would lead to even larger increases in prices. On the other hand, the relative stability of the nominal, specific excises currently levied in the United States has contributed substantially to reductions in the real prices of alcoholic beverages.

### Other Policies Affecting Alcoholic-Beverage Prices

In addition to tax-related polices, several other regulations also may directly or indirectly affect the prices of alcoholic beverages. These options include, but are not limited to, regulations on wholesale and retail distribution, bans on price-related promotions, and (targeted) minimum-pricing policies. However, the empirical evidence on the impacts of these policies is very limited.

After the repeal of Prohibition through the 21st Amendment to the U.S. Constitution, States imposed differing degrees of control over various aspects of the wholesale and retail distribution system of alcoholic beverages as part of the creation of a “three-tier system” for alcohol distribution. Thus, some States now monopolize the retail sale (for off-premises consumption) and wholesale sale (including sales to outlets licensed to sell for on-premises consumption) of some alcoholic beverages (most often distilled spirits and, in some States, wine). These are known as “monopoly” or “control” States. Other States, however, do not directly control the distribution system for alcoholic beverages but instead have adopted a mixed set of regulations that influence the extent of competition in alcoholic-beverage markets. These regulations include the licensing of retailers and wholesalers, restrictions on the distribution of alcoholic beverages (e.g., at-rest laws, primary-source laws, direct-shipping laws, and reciprocity laws), and/or the adoption of exclusive territory policies that grant monopoly power over a particular geographic area to a specific distributor. These States also are referred to as “license States.”

As a consequence of these policies, competition is reduced at some point in the local supply chain for alcoholic beverages, and economic theory predicts that prices would be higher in such less competitive markets. However, the empirical evidence on the impact of these policies on prices is limited and, at times, inconsistent. For example, Nelson (1990) found that the prices were slightly higher in monopoly States compared with prices in license States, whereas MacDonald (1986) concluded the opposite. Several studies focusing on the impact of exclusive territory policies for beer distribution determined that these policies did result in higher beer prices (Culbertson 1989; Culbertson and Bradford 1991; Jordan and Jaffee 1987; Sass and Saurman 1993, 1996).

In addition to indirectly influencing the prices for alcoholic beverages by modulating the extent of competition in the market, States also have the option to regulate prices directly by restraining price-related promotions (e.g., by limiting promotions such as “happy hour” specials, the selling of beer by the pitcher, or the free sampling of alcoholic beverages) or imposing minimum-pricing policies. However, research on the impact of these policies on the prices drinkers pay for alcoholic beverages, drinking behaviors, and associated consequences is almost nonexistent (Chaloupka 2004). One exception is a study by Meier and colleagues (2010), who examined the impact of various policy options—including minimum-pricing policies (general or targeted), bans of below-cost selling, and restrictions on price-based promotions—on alcohol prices and subsequent alcohol consumption among different population subgroups in England. The study demonstrated that the heterogeneity and complexity among alcohol users were important factors when considering policy options. The results also suggested that compared with general price increases, minimum-pricing policies might affect harmful drinkers proportionately more, but young hazardous drinkers less, than drinkers in general.^5^

In recent years, some large alcoholic-beverage retailers have filed a couple of lawsuits against these non–tax-related State regulations over their perceived anticompetitive effects. For example, in Maryland, a large regional retailer has challenged the State’s ban on quantity discounts for wine and spirits at the wholesale level and the related price post-and-hold requirements (TFWS vs. Schaefer et al.) (Chaloupka 2010). Similarly, in Washington, a large national retailer has challenged a broader set of policies regulating wholesale distribution that includes minimum mark-up, cash payment, and direct-delivery provisions in addition to the State’s ban on quantity discounts and related post-and-hold requirements (Costco v. Hoen et al.) (Chaloupka 2010). In both cases, the States have adopted a defense based on the 21st Amendment to oppose these legal challenges, arguing that the regulations were consistent with the State public health interests by reducing excessive drinking. However, the States’ ability to defend themselves against such legal challenges has been hampered by limited published evidence on the effects of these policies on alcohol prices, consumption, and related consequences of drinking.

In summary, non–tax-related State regulations that may directly or indirectly affect alcohol prices, drinking, and its consequences have been eroded in recent years, in part because of limited empirical evidence on their impact. The resulting further relaxation of these types of State regulations can contribute substantially to additional reductions in the real prices of alcoholic beverages, increased drinking, and greater alcohol-related harm. Studies addressing these issues are warranted and will greatly enhance understanding of the impact of changes in these policies on prices of alcoholic beverages.

## The Effects of Prices on Alcohol Use and Related Consequences

Alcohol consumption potentially is addictive, at least for some drinkers. However, numerous studies over the last two decades using a variety of econometric and statistical methods and different types of data have confirmed that higher prices substantially can reduce alcohol use (and abuse) and related adverse consequences even among heavier drinkers. This section provides a review of general findings from these studies.

### Effects of Price on Alcohol Use and Abuse

Extensive literature exists of studies assessing the effects of changes in price on the levels of alcohol use and abuse. Because of the vast amount of literature available, this review focuses mainly on findings from published meta-analyses and reviews and only uses individual studies for illustration, where relevant.

One commonly used concept in economic studies exploring the impacts of prices on drinking behaviors is termed the price elasticity of the demand for alcohol. This variable represents the percentage change in the consumption of alcoholic beverages that occurs when the price increases by 1 percent, holding other factors constant. Thus, a price elasticity of the demand of, for example, −0.3 indicates that a 1 percent increase in price results in a 0.3 percent decrease in consumption. An early review of studies based on aggregated sales data from the United States and other countries indicated that the price elasticities of the demand for beer, wine, and distilled spirits were −0.3, −1.0, and −1.5, respectively (Leung and Phelps 1993), indicating that consumption of distilled spirits was more responsive to price increases than beer consumption. More recently, Wagenaar and colleagues (2009) reported that average price elasticities from 40 studies based on aggregated sales data were −0.17 for beer, −0.30 for wine, and −0.29 for spirits, whereas Elder and colleagues (2010) determined that median elasticities ranged from −0.51 to −0.90 in the 38 articles they reviewed.

Compared with studies based on aggregated data (mainly State alcohol-beverage sales), studies using individual self-reported alcohol consumption generally found that the demand for alcohol was more responsive to price (Chaloupka et al. 2002; Fogarty 2006; Gallet 2007; Wagenaar et al. 2009). For example, Wagenaar and colleagues (2009), on the basis of an analysis of 112 studies using various types of data, indicated that the average elasticities were −0.46 for beer, −0.69 for wine, and −0.80 for spirits. Likewise, Gallet (2007) showed that among 300 estimates, the median elasticities were −0.36, −0.70, and −0.68 for beer, wine, and spirits, respectively.

Because alcohol consumption is addictive in the sense that increases in past consumption cause current consumption to rise, the immediate reduction in alcohol consumption associated with price increases in the current time period (in the short run) may lead to future decline in alcohol consumption in the future (in the long run). Studies have suggested that the demand for alcoholic beverages seems to be more responsive to changes in prices over the long run than over the short run. For example, using individual-level data from the Monitoring the Future study, Grossman and colleagues (1998) determined that that the average long-run price elasticity for alcohol was −0.65, whereas the short-run elasticity was −0.41. This finding was confirmed by subsequent studies. For example, the review by Gallet (2007) revealed that median price elasticities for all beverages were −0.52 in the short run and −0.82 in the long run.

Many studies have shown that not only social drinking but also abusive drinking (i.e., alcohol dependence and/or binge drinking) is responsive to price, although apparently to a lesser extent. Although the findings are mixed about the relative price sensitivity of abusive and nonabusive drinkers, most studies have reported that heavy/frequent drinkers normally are less responsive to price changes than light/infrequent drinkers. Thus, the estimated price elasticity of abusive drinkers ranges from −0.01 to −0.10 (Chaloupka and Laixuthai 1997; Chaloupka and Wechsler 1996; Cook and Moore 2000; French et al. 2006; Keng and Huffman 2007; Kenkel 1993, 1996; Laixuthai and Chaloupka 1993, Sloan et al. 1995; Stout et al. 2000). A more recent review of 10 studies on the effects of alcohol prices on various measures of alcohol abuse indicated that the average price elasticity was −0.28 (Wagenaar et al. 2009).

### Effects of Price on Alcohol Use Among Youth and Young Adults

Several studies have addressed the effects of alcohol prices on the drinking behaviors of youths and young adults. This population is of particular relevance because they exhibit relatively high levels of binge drinking and of alcohol-related problems; moreover, there seems to be great potential for using tax and price policies to prevent underage drinking. Using data from early waves of National Health and Nutrition Examination Surveys, Grossman and colleagues (1987) and Coate and Grossman (1988) were the first to examine the impact of price on alcohol use among adolescents. These investigators found that price increases led to larger reductions in the fractions of heavy and fairly heavy adolescent drinkers than in the fraction of light drinkers. Most subsequent studies confirmed this finding (Cook and Moore 2001; Laixuthai and Chaloupka 1993; Young and Bielinska-Kwapisz 2006). Moreover, Laixuthai and Chaloupka (1993) reported that younger adolescent drinkers were more price sensitive than older adolescent drinkers, which is a particularly important finding because Cook and Moore (2001) provided evidence supporting the notion that drinking habits are formed at young ages.

Using data from the Harvard College Alcohol Survey, Chaloupka and Wechsler (1996) conducted the first study investigating the impact of prices on the drinking behavior among young adults. These investigators detected price effects only among underage female students—a finding that they attributed to limitations in the available data for prices. In a subsequent study using data from a longer panel of the Harvard College Alcohol Survey and better measures of price, Williams and colleagues (2005) showed that increases in prices led to reductions in alcohol use among both moderate and heavy college drinkers. More importantly, the impact was the same for both groups, indicating that price effects were independent of drinking intensity. In another study using longitudinal data from the Monitoring the Future surveys in models that explicitly accounted for the addictive nature of alcohol use, Grossman and colleagues (1998) also measured a causal effect of prices on alcohol consumption among young adults. These conclusions are consistent with those from numerous recent studies, all of which have demonstrated an inverse relationship between prices and the consumption of alcoholic beverages among adolescents and youth (Bloomfield et al. 2009; Hollingworth et al. 2006; Kuo et al. 2003*a*, *b*; O’Mara et al. 2009; Wechsler et al. 2000). For example, Kuo and colleagues (2003*b*) found that low sale prices and special promotions resulting in reduced prices were associated with higher binge-drinking rates among college students. Likewise, using data collected from patrons immediately after drinking in on-premise establishments, instead of from retrospective surveys (i.e., event-level data), O’Mara and colleagues (2009) demonstrated that increases in alcohol prices were accompanied by reduced consumption among college students.

In summary, nearly all studies investigating the effects of price on drinking, both in the general population and in population subgroups (e.g., heavier drinkers or youth and young adults), have identified a downward-sloping demand curve, indicating that the consumption of alcoholic beverages would be reduced if prices were raised. The impact of such measures seems to be larger in the long run than in the short run and tends to be particularly strong for adolescents and young adults (e.g., Cook and Moore 2001; Elder et al. 2010; Gallet 2007; Grossman et al. 1998; Kuo et al. 2003*a*, *b*; Laixuthai and Chaloupka 1993). These conclusions have important policy implications and suggest that raising prices of alcoholic beverages not only postpones drinking initiation and addiction formation among adolescents and young adults but also reduces heavy or chronic alcohol use among adults.

### Effects of Prices on Consequences of Alcohol Abuse

Many studies have shown that in addition to reducing alcohol use and abuse, increased prices also decrease the adverse consequences resulting from alcohol use and abuse. This section provides a brief review of findings from the literature in this area.

#### Drinking and Driving

Most studies in this field have used fatal or nonfatal motor-vehicle crashes as a proxy for drinking and driving, because alcohol frequently is involved in these crashes. Early studies showed that increases in beer taxes significantly reduced fatal motor-vehicle crash rates, particularly among youth (Cook 1981; Saffer and Grossman 1987*a*, *b*). Subsequent studies using updated panel data and robust specifications consistently confirmed the conclusion that higher taxes and prices significantly reduce drinking and driving (Chaloupka et al. 1993; Ruhm 1996; Sloan et al. 1994).

A few studies, however, have generated contradictory results. For example, Mast and colleagues (1999) replicated the analysis by Chaloupka and colleagues (1993) but used data obtained from 1984 to 1992, rather than from 1982 to 1988. These investigators failed to detect a significant relationship between tax rates and drinking-and-driving rates. Likewise, Dee (1999) and Young and Likens (2000) found that there was little relationship between beer taxes and motor-vehicle fatalities, and the significance of the relationship was very sensitive to the specification of regression models. As one explanation for these inconsistencies, Mast and colleagues (1999) argued that the minimum legal drinking ages had been higher in the later years of data collection, which means that beer taxes had become a smaller part of the “full price” of teen drinking. Another potential explanation comes from the distinction between alcohol-related and non–alcohol-related traffic fatalities. Thus, studies that do not separate alcohol-related fatal crashes from non–alcohol-related crashes may come to different conclusions than studies that do make this distinction (Elder et al. 2010; Fell et al. 2009; Miron et al. 2008). In their recent review, Elder and colleagues (2010) concluded that studies that assessed fatalities more directly attributable to alcohol consumption (e.g, nighttime fatal traffic crashes) usually found larger price effects than did studies for which the relationship to alcohol consumption was less direct (e.g., studies assessing all crash fatalities).

Most recent research, however, consistently has documented an inverse association between prices (i.e., beer taxes) and traffic fatalities (Elder et al. 2010; Makela and Osterberg 2009; McCarthy 2003; Ponicki et al. 2007; Wagenaar et al. 2010; Young and Bielinska-Kwapisz 2006). For example, using alcohol taxes as instrumental variables to correct measurement errors in price data, Young and Bielinska-Kwapisz (2006) found that higher prices of alcoholic beverages significantly reduced motor-vehicle fatalities. Elder and colleagues (2010), in a review of 11 studies, concluded that the relationship between alcohol prices or taxes and injuries and deaths from motor-vehicle crashes generally was significant and of a comparable magnitude to the relationship between these variables and alcohol consumption. Finally, a meta-analysis of 34 independent estimates also confirmed the statistically significant inverse association (Wagenaar et al. 2010).

A few studies that used self-reported drinking-and-driving measures likewise concluded that higher prices or taxes would significantly reduce the probability of nonfatal crashes, particularly among youth (e.g., Chaloupka and Leixuthai 1997; Kenkel 1993).

#### Crimes

Several studies have examined the relationship between prices of alcoholic beverages and the prevalence of violence and other related crimes. Early studies in this field reached slightly differing conclusions. For example, Cook and Moore (1993*a*) found that higher beer taxes would reduce the prevalence of rape and robbery but not of homicides and assaults, whereas Sloan and colleagues (1994) noted that homicide deaths declined with increases in alcohol prices. Later, Saffer (2001) suggested that raising beer taxes led to significant reductions in the involvement in crimes, including arrests, property crime, property damage, physical violence, and drug selling; moreover, this effect was particularly pronounced for adolescents. Most recently, researchers have demonstrated that declining alcohol prices in England and Wales were associated with substantial increases in violence-related injuries and trauma services (Matthews et al. 2006; Sivarajasingham et al. 2006). Likewise, Makela and Osterberg (2009) documented that the 2004 tax cuts in Finland were followed by temporary increases in manslaughters and murders.

A series of articles by Markowitz and Grossman focused on the impact of alcohol prices on family violence and violent behavior among youth. These investigators have shown that increases in beer taxes are associated with reductions in child abuse, particularly child abuse committed by women (Markowitz and Grossman 1998, 2000). Their subsequent studies provided evidence that higher alcoholic-beverage prices also led to reductions in violence and other delinquent behaviors among college and high-school students, as well as to a drop in severe violence committed by husbands to their wives (Grossman and Markowitz 2001; Markowitz 2000, 2001). In another study using individual-level data from the National Crime Victimization Surveys, Markowitz (2005) demonstrated that higher beer taxes reduced the probability of assault.

#### Liver Cirrhosis and Other Alcohol-Related Mortality

A relatively small but growing number of studies have examined the impact of alcoholic-beverage prices (or taxes) on alcohol-related mortality, including liver cirrhosis mortality, and suicides. Early studies suggested that cirrhosis mortality rates immediately responded to price changes and that the long-run effect could be even greater (Cook 1981; Cook and Tauchen 1982; Grossman 1993). For example, estimates have shown that, in the long run, a 10 percent increase in prices would lead to 8.3 to 12.8 percent reductions in the cirrhosis mortality rate (Grossman 1993). More recent studies exploring the effects of the reductions in alcoholic beverage taxes in Finland have reached the same conclusion (Herttua et al. 2008; Makela and Osterberg 2009), reporting dramatic increases in liver cirrhosis mortality for both men and women immediately after the tax cuts. Moreover, Herttua and colleagues (2008) found that such impacts were particularly pronounced among older men (ages 55–59) and women (ages 50–54), as well as among people with low socioeconomic status. Recent evidence linking tax rates and alcohol-related mortality also has come from the United States. Using death-certificate data and a quasiexperimental research design, Wagenaar and colleagues (2009) noted that increases in alcohol excise taxes in Alaska were associated with immediate and sustained reductions in alcohol-related disease mortality. Likewise, Wagenaar and colleagues (2010) presented evidence for the significant association between alcohol prices and alcohol-related disease in another review.

However, there are exceptions to these findings. For example, Sloan and colleagues (1994) did not detect a significant impact of beer prices on cirrhosis mortality but instead found strong price effects on suicides and other accidental death.^6^ Other studies have confirmed the latter finding, suggesting that higher beer prices (or taxes) reduced suicidal behavior among adolescents, particularly among male subjects (Chatterji et al. 2004; Markowitz et al. 2003). On the other hand, Birckmayer and Hemenway (1999) failed to find an association between beer tax and suicides among youth. Finally, Yamasaki and colleagues (2005) demonstrated that increases in alcohol taxes correlated significantly with suicides in male but not in female subjects. These inconsistencies regarding the relationship between prices of alcoholic beverages and suicide may result from measurement errors in the dependent variable, because not all suicides were alcohol related. In fact, according to a meta-analysis by Smith and colleagues (1999), blood alcohol concentrations (BACs) indicative of intoxication (BAC more than or equal to 100 mg/dL) were found in a much smaller percentage of suicide cases (i.e., 22.7 percent) than of homicide cases (31.5 percent). In general, a meta-analysis based on 11 independent estimates from four studies indicated that the impact of prices of alcoholic beverages on suicide only was marginally significant (Wagenaar et al. 2010).

#### Risky Sex, Sexually Transmitted Diseases, and Abortion

Over the past decade, more research has assessed the impact of alcoholic beverage prices on reproductive issues, including risky sexual behaviors, sexually transmitted diseases, and abortion, especially among teenagers. Using State-level data, Chesson and colleagues (2000) demonstrated that higher taxes on beer and spirits significantly reduced the prevalence of gonorrhea and syphilis. Similar results were obtained using gonorrhea rates between 1981 and 2001 among adolescents and youth ages 15–24 (Grossman et al. 2005). A subsequent study also concluded that gonorrhea and acquired immune deficiency syndrome (AIDS) rates could be reduced with higher beer taxes (Markowitz et al. 2005). More recently, an analysis of data obtained across provinces in Canada demonstrated that higher beer prices were significantly associated with reductions in both gonorrhea and chlamydia rates, with price elasticities ranging from −0.7 to −0.9 (Sen and Luong 2008). Finally, based on a review of four studies, Wagenaar and colleagues (2010) concluded that an inverse relationship existed between alcohol taxes or prices and rates of sexually transmitted disease and risky sexual behaviors.

An inverse relationship also has been identified between beer taxes and abortion rates among teenagers (Sen 2003). This observation has been confirmed by individual-level data from the Youth Risk Behavior Surveys, which demonstrated that increases in beer taxes promoted the use of condoms and other birth-control methods among teenagers (Grossman and Markowitz 2005).

#### School Performance

Several studies have explored the effects of prices of alcoholic beverages on schooling, using both extensive (i.e., education attainment) and intensive (i.e., performance in school) measures. Two analyses using data from the National Longitudinal Survey of Youth have shown that higher alcohol taxes led to increases in high-school graduation and post–high-school education attainment (Cook and Moore 1993*b*; Yamada et al. 1996). Likewise, evaluations of detailed information on school performance of college students from the Harvard College Alcohol Survey demonstrated that increases in prices (including those from limiting promotions that lowered prices of alcoholic beverages) led to improvements in grade-point averages as well as significant reductions in the likelihood of missing classes and the probability of falling behind (Powell et al. 2002; William et al. 2002).

Taken together, with few exceptions, studies have reported consistent evidence that higher prices (and/or taxes) of alcoholic beverages are negatively associated with adverse consequences of drinking. Most existing studies focused on the association of taxes/prices of alcoholic beverages and undesirable consequences related to excessive drinking. However, a direct demonstration that changes in alcohol prices cause changes in adverse consequences (rather than just being associated with them) still is lacking and should be the focus of future studies. Additional investigations also are warranted in areas with inadequate and sometimes inconsistent evidence. For example, whereas numerous studies have evaluated the effects of price on drinking and driving, only few analyses have examined the impact of taxes/prices of alcoholic beverages on suicides, risky sexual behaviors (and abortions) among adults, and school performance. In addition, as discussed earlier, very limited attention has been paid to the effect of non–tax-related policies that may affect prices of alcoholic beverages and, as a result, consequences of alcohol abuse. Studies in these areas will greatly expand the existing knowledge on different policy options.

## Discussion and Conclusions

A large and growing literature has explored the impact of prices of alcoholic beverages on alcohol use and abuse as well as related adverse consequences. The vast majority of these studies provide strong evidence supporting efforts to raise Federal or State taxes to promote public health by reducing drinking, including abusive drinking and its consequences. More importantly, these studies clearly indicate that adolescents and youth are more responsive to changes in prices than the general population, implying that the implementation of tax policies not only could produce immediate public health benefits but achieve even greater success in the long run. From a public finance perspective, raising alcohol taxes also is among the most cost-effective instruments to reduce harm and promote public health (Anderson et al. 2009).

The economic costs that result from alcohol use and abuse provide another strong argument for raising excise taxes on alcoholic beverages. In 2006, the Federal Government received about $9.2 billion from alcohol excise taxes, with State governments collecting another $4.9 billion. By comparison, the economic costs of excessive drinking in 2006 were estimated at $223.5 billion (Bouchery et al. 2011). Thus, the economic costs of alcohol far exceed the excise tax revenue from alcoholic beverages. In other words, people who do not use alcohol have been subsidizing alcohol users, especially the top 20 percent of drinkers who consumed approximately 85 percent of all alcoholic beverages (Rogers and Greenfield 1999).

In addition, several studies consistently have demonstrated that current excise taxes are substantially below the “optimal” level when one considers the external costs (i.e., costs borne by nondrinkers or moderate drinkers) of alcohol use. Pogue and Sgontz (1989) showed that the “best-guess” estimate based on their model was 51 percent of the net price of the beverage (i.e., price excluding tax), whereas Kenkel (1996) estimated the optimal tax should be around 106 percent of the net price. Two other studies (Manning et al. 1989; Saffer and Chaloupka 1994) suggested that the excise tax rates during their study period would have had to be doubled to reach the optimal level. Given that State and Federal taxes generally have not kept pace with inflation since these studies were done (see figures 1 and 2), the “optimal” tax likely would have to be even higher today.

To date, evidence such as the findings presented here has had little impact on public policy, with the Federal Government and most State governments allowing the inflation-adjusted value of their alcoholic beverage taxes to fall as demonstrated by the infrequent and modest increases in these taxes. In contrast, the Federal Government and many State governments have adopted several large increases in taxes on cigarettes and other tobacco products, at least in part to promote public health by reducing tobacco use. Similarly, indexing alcohol excise taxes to inflation in order to prevent substantial reductions in real tax rates would help ensure that higher taxes have a sustained impact on drinking and its consequences. As the studies presented in this review demonstrate, sizable increases in alcoholic beverage taxes can be a highly effective option for reducing the health, economic, and social consequences of alcohol use and abuse.

## Footnotes

1 Dram shop laws govern the liability of establishments that serve alcohol. In particular, they state that these establishments are liable if they serve alcohol to obviously intoxicated patrons or to minors who then cause harm to third parties as a result of alcohol-related car crashes and similar accidents.

2 For a detailed historical record of Federal tax rates for alcoholic beverages, see the Web site of the Alcohol and Tobacco Tax and Trade Bureau (TTB), http://www.ttb.gov/tax_audit/94a01_4.shtml.

3 License States are States that adopt a mixed set of regulations to influence the extent of competition in alcoholic beverage markets rather than directly control distribution and sale of these beverages. Please refer to the section, Other Polices Affecting Alcoholic-Beverage Prices, for a detailed discussion on license States.

4 The beer tax has been used as an example, because beer is the most widely consumed alcoholic beverage in the United States and nearly all States apply specific taxes to beer.

5 The terms moderate, hazardous, and harmful drinkers relate to how much people drink and are the standard definitions used by the Office for National Statistics in the United Kingdom. Moderate drinkers are those who drink no more than 21 units per week for men or 14 units per week for women. (Units are based on U.K. standards, with one unit corresponding to 10 mL pure ethanol.) Hazardous drinkers are those who drink above these guidelines but do not consume more than 50 units per week for men or 35 units per week for women. Harmful drinkers are men who drink more than 50 units per week or women who drink more than 35 units per week—a level of drinking that has been associated with a high risk of both acute and chronic harm.

6 Ohsfeldt and Morrisey (1997) also found evidence that higher beer taxes led to significantly fewer nonfatal injuries in the workplace
